# Etiology and Risk Factors for Rheumatoid Arthritis: A State-of-the-Art Review

**DOI:** 10.3389/fmed.2021.689698

**Published:** 2021-11-26

**Authors:** Vasco C. Romão, João Eurico Fonseca

**Affiliations:** ^1^Rheumatology Department, Hospital de Santa Maria, Centro Hospitalar Universitário Lisboa Norte, Lisbon Academic Medical Centre and European Reference Network on Rare Connective Tissue and Musculoskeletal Diseases Network (ERN-ReCONNET), Lisbon, Portugal; ^2^Rheumatology Research Unit, Instituto de Medicina Molecular João Lobo Antunes, Faculdade de Medicina, Universidade de Lisboa, Lisbon, Portugal

**Keywords:** rheumatoid arthritis, etiology, risk factors, pathogenesis, pre-rheumatoid arthritis, pre-RA

## Abstract

Rheumatoid arthritis (RA) is the most common systemic inflammatory rheumatic disease. It is associated with significant burden at the patient and societal level. Extensive efforts have been devoted to identifying a potential cause for the development of RA. Epidemiological studies have thoroughly investigated the association of several factors with the risk and course of RA. Although a precise etiology remains elusive, the current understanding is that RA is a multifactorial disease, wherein complex interactions between host and environmental factors determine the overall risk of disease susceptibility, persistence and severity. Risk factors related to the host that have been associated with RA development may be divided into genetic; epigenetic; hormonal, reproductive and neuroendocrine; and comorbid host factors. In turn, environmental risk factors include smoking and other airborne exposures; microbiota and infectious agents; diet; and socioeconomic factors. In the present narrative review, aimed at clinicians and researchers in the field of RA, we provide a state-of-the-art overview of the current knowledge on this topic, focusing on recent progresses that have improved our comprehension of disease risk and development.

## Introduction

Rheumatoid arthritis (RA) is a chronic immune-mediated multisystemic disease that mainly localizes to the joints. It is the most common systemic inflammatory rheumatic disease (1, 2) and it is associated with considerable morbidity and disability, as well as increased mortality (3). In the last decades, the prognosis of RA patients has been dramatically improved by the expansion of knowledge on the etiology and pathophysiology of the disease that paved the way for the development of a number of currently available effective drugs (4). However, despite these advances, there is still a substantial gap in the management of the disease, with many patients failing to attain profound and sustained clinical responses, ultimately demonstrating modest long-term outcomes. Even more strikingly, the actual impact on the prevention or delay of the disease in subjects at high-risk has overall been marginal.

Extensive efforts have been devoted in the last decades to investigate the epidemiological association of several factors with the risk of developing RA, as well as its course and prognosis. Tremendous progress has, nonetheless, been made. Although a precise etiology is yet to be determined, it is apparent that RA is a multifactorial disease, with a complex interplay between the host and the environment determining the overall risk of disease susceptibility, persistence and severity (5, 6). In fact, this intricacy is well-illustrated in the European League Against Rheumatism (EULAR) definition of the stages that unfold until the development of RA (Box 1) (7). The insights from the wealth of epidemiological studies available, though not directly proving causality, are important for understanding the etiology and pathogenesis of RA. Additionally, they can provide support to patient advice on modifiable risk factors.

Box 1 **Stages preceding the development of RA as proposed by EULAR.**

- Individuals may go through different phases prior to RA onset:
  a. Genetic risk factors for RA
  b. Environmental risk factors for RA
  c. Systemic autoimmunity associated with RA
  d. Symptoms without clinical arthritis
  e. Unclassified arthritis
  f. RA
- *a*. to *e*. may be present simultaneously at a given moment (e.g., a+c+d)
- ‘Pre-RA' should only be applied retrospectively to patients with RA, do describe a phase where any of *a*. to *e*. are present, individually or in combination.

Adapted from Gerlag et al. Ann Rheum Dis 2012 (7).

**Figure 1:**
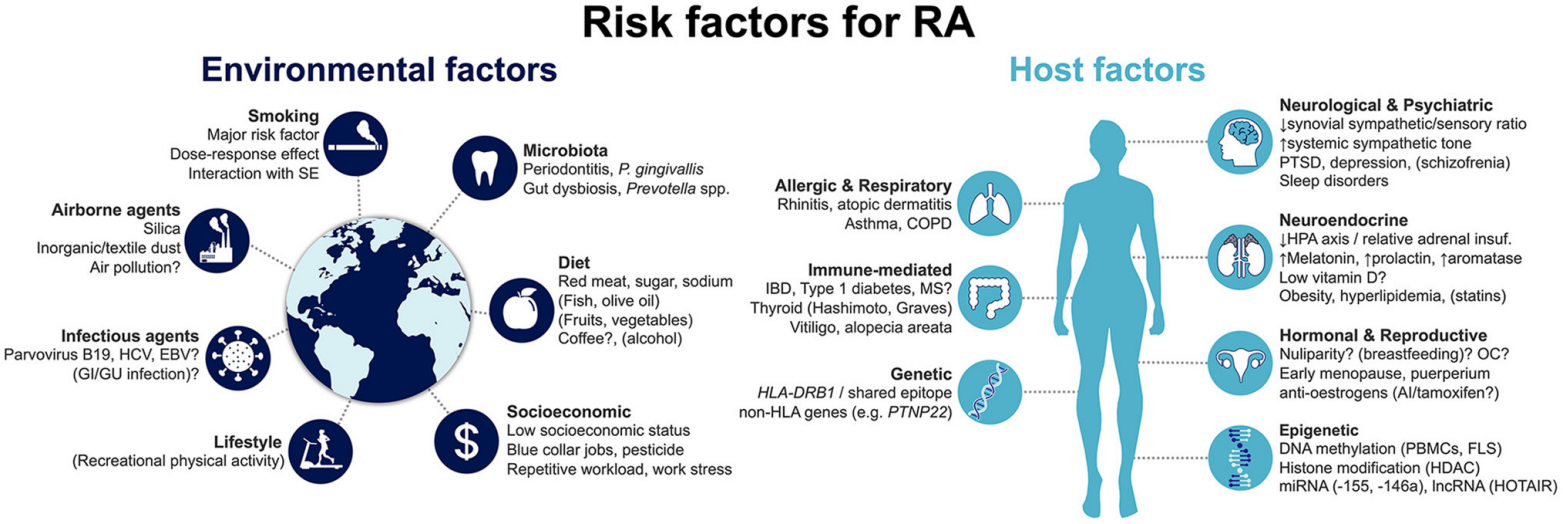
Summary of risk factors for the development of rheumatoid arthritis. Factors that are associated with decreased risk are represented in brackets. Factors for which evidence is conflicting and uncertainty remains are followed by a question mark. AI, aromatase inhibitors; COPD, chronic obstructive pulmonary disease; EBV, Epstein-Barr virus; FLS, fibroblast-like synoviocyte: GI, gastrointestinal; GU, genitourinary; HCV, hepatitis C virus; HDAC, histone deacetylases; HLA, human leukocyte antigen; HPA, hypothalamic-pituitary-adrenal; IBD, inflammatory bowel disease; lncRNA, long non-coding RNA; miRNA, micro RNA; MS, multiple sclerosis; OC, oral contraceptives; PBMCs, peripheral blood mononuclear cells; PTSD, post-traumatic stress disorder; SE, shared epitope.

Risk factors for developing RA can be generically divided into host- and environment-related (Figure 1). Host factors that have been associated with RA development may be further grouped into genetic; epigenetic; hormonal, reproductive and neuroendocrine; and comorbid host factors. In turn, environmental risk factors include smoking and other airborne exposures; microbiota and infectious agents; diet; and socioeconomic factors. Herein, we provide a state-of-the-art overview of the current knowledge on this topic, aimed at clinicians and researchers in the field of RA. We specifically focus on recent progresses that have improved our comprehension of disease risk and development.

## Host Factors

As with many other immune-mediated diseases, the host is closely linked to the risk for developing RA (Figure 1). This includes, first and foremost, genetic factors, which account for a major proportion of disease risk. More recently, epigenetic mechanisms have been identified to be directly involved in RA pathogenesis, modulating the risk of disease development. Notably, they can be influenced by the environment, linking extrinsic and intrinsic factors. Hormonal, reproductive and neuroendocrine factors have long been proposed as contributing to RA, given the observed female preponderance of the disease. Finally, a number of concomitant pre-existing conditions have been proposed to increase the risk of incident RA. We detail the available evidence concerning each of these groups in the following sections.

### Genetic Factors

Data supporting a genetic component in RA first arose from familial and twin studies. In fact, the risk of a monozygotic twin of an RA patient for developing RA is 9–15%, which is up to 4-fold that seen for dizygotic twins, and much higher than the general population [relative risk (RR) 25–35] (8–10). Likewise, first-degree relatives have a RR of RA that varies from 2 to 5 and is similar in men and women (11–14). In addition, the risk of RA is also increased by 1.5–3-fold in offspring of parents with other immune-mediated inflammatory diseases, such as systemic lupus erythematosus, Sjögren's syndrome, ankylosing spondylitis, or Hashimoto thyroiditis (12). These studies have allowed the estimation of the heritability of RA, that is, the quantification of the genetic contribution of the disease, which was found to be 50–65% (15). Interestingly, it has been recently shown to be higher in ACPA-positive RA (50%), compared to ACPA-negative disease (20%) (11).

Currently, over 100 loci have been associated with increased RA risk in trans-ethnic populations (16, 17). Due to its importance for the immune system, the **major histocompatibility complex (MHC**) locus was the first to be identified and remains the most studied region, accounting for around a third of the disease genetic susceptibility (18). Certain human leukocyte antigen (HLA) loci such as *HLA-DRB1* have been found to be strongly associated with RA in most populations (19). However, the risk varies according to specific alleles and ancestry, being higher for HLA-DRB1^*^0101/^*^0401/^*^0404 in Caucasians, HLA-DRB1^*^0405/^*^0901 in Asians, and HLA-DRB1^*^1402 in Native American Indians (19, 20). A major breakthrough was the identification of a sequence of five amino acids in residues 70–74 (QKRAA, QRRAA, RRRAA) in the third hypervariable region of the DRß1-chain, encoded by the *HLA-DRB1* gene, that was highly preserved in risk alleles for RA and was therefore named shared epitope (SE) (19, 21). The SE hypothesis proposed that this given sequence enabled binding of a specific peptide to the HLA molecule of antigen-presenting cells (APCs), which was recognized by T cells, eventually leading to an autoimmune response. While such an antigen has not been unequivocally identified so far, and the role of the SE-containing alleles seems less important in some populations (19), the SE hypothesis was vital in providing an etiological support to the aforementioned epidemiological observations. Yet, this theory fails to explain the differential risk conferred by SE alleles (higher with ^*^0401 and ^*^0405 vs. ^*^0101 and ^*^0404, respectively) suggesting that other genetic factors may be involved (22, 23). Moreover, the presence of the SE is more strongly associated with ACPA-positive than ACPA-negative RA (23–25), and has been linked with more severe disease (23, 26), extra-articular manifestations (23, 27) and radiographic damage (28).

Subsequent studies have complemented the SE hypothesis and determined that almost all of the RA risk conferred by the MHC region is explained by six amino acids in four HLA molecules: HLA-DRB1 (positions 11/13, 71 and 74), HLA-B (position 9), HLA-DPB1 (position 9), and HLA-A (position 77) (17, 29, 30). It should be noted that despite most of these amino acids being located outside the SE region, all are found in the peptide binding grooves of HLA, strengthening the importance of antigen presentation to T cells [both cluster of differentiation (CD) 4^+^ and CD8^+^] for the pathogenesis of RA. Importantly, these and other recent studies also confirmed the association of *HLA* genes, including *HLA-DRB1* SE alleles, with seronegative RA, although with a smaller effect size and a differential pattern from ACPA-positive disease (17, 31–33). Finally, it was proposed that a 16-category hierarchy was adopted for RA susceptibility, instead of the SE-positive/negative approach, based on the positions 11/13, 71 and 74 of the *HLA-DRB1* alleles (29). These categories were also recently confirmed to be associated with radiographic damage and mortality (34, 35).

Given that only 30% of the genetic risk for RA is explained by the MHC region, significant effort has been devoted to studying **non-HLA genes**. This has been done through candidate gene association studies or large-scale genome-wide association studies. Until now, more than 100 risk loci have been validated across multiple populations (16, 17, 36, 37). Some important conclusions have emerged: (i) each allele confers a small risk increase [odds ratio (OR) usually <1.5–2.0] and multiple susceptibility genes interact to determine the occurrence of disease; (ii) the identified genes have contributed to better understand the mechanisms of disease, as most are directly linked to the immune system; (iii) only 20% of risk loci include coding variants, with the remaining 80% being linked to other processes such as gene expression regulation, non-coding ribonucleic acids (RNAs) or post-transcriptional changes; (iv) despite all of the advances, non-HLA alleles explain only 5–6% of genetic variation (16, 17, 36, 37).

Some of the most studied non-HLA genetic variants are related to immune cell function and therefore deserve mention. A single nucleotide polymorphism (SNP; R620W) in the *PTPN22* gene, which encodes a protein tyrosine phosphatase involved in antigen receptor signaling of B and T cells, has been the first to be widely replicated and remains the second stronger genetic risk factor for RA, with an OR just under 2 (38). This gain-of-function variant alters T and B cells activation thresholds, leading to changes in clonal selection and emergence of autoreactive cells (38). The R620W SNP has only been associated with seropositive RA, and support for this observation comes from studies showing that it also results in enhanced peptide citrullination (33, 39). Interestingly, this SNP is absent in East Asian (e.g., Japanese) populations, that instead present common genetic variants of *PADI4*, a gene encoding peptidyl-arginine deiminase (PAD, a peptide citrullination enzyme), that are associated with increased risk of RA (OR 1.31/allele copy) (38, 40, 41). Other loci and genes involved in inflammatory pathways that have been implicated in RA with a modest effect size include *CTLA4* (negative regulator of T cell activation) (41), *STAT4* (transcription factor involved in intracellular cytokine signaling) (42), *TNFAIP3* [inhibitor of nuclear factor κ-light chain enhancer of activated B cells (NF-κB) signaling; it is required for termination of tumor necrosis factor (TNF)-induced signals] (43), *TRAF1-C5* (locus including *TRAF1*, which encodes a negative regulator of intracellular TNF signaling that binds to TNFAIP3, and *C5*, which encodes complement factor C5) (44), *IL2/21* [encoding interleukin (IL)-2 and IL-21] (45), *CD40* (surface receptor present in various immune cells, including B cells, where it is crucial for B-T cell interaction) (46), *IL2RA/IL2RB* (encoding IL-2 receptor alpha and beta chains, respectively), (47, 48) *IL6R* [encoding the IL-6 receptor (IL-6R)] (49) or *CCL21* (lymphocyte chemokine) (46), among many others (16, 50, 51). While some genes, such as *STAT4* and *CTLA4*, have not been shown to be of genome-wide significance, this is a rapidly expanding field with large-scale studies identifying or confirming novel associations, most often in loci associated with immune function and regulation (17, 49–51). In addition, novel genes studied in other fields such as oncology, are emerging in RA. For instance, the human *RNASET2* tumor suppressor gene has been associated with RA development in Asian populations (52). This gene encodes for ribonuclease T2, an enzyme implicated in cancer development, and recently shown to regulate the innate immune response (e.g., macrophage function), which is implicated in RA development (53, 54). Importantly, besides disease susceptibility, some of the non-HLA genes have also been associated with severity (55, 56) and differences in seropositive and seronegative RA (31, 57).

### Epigenetic Factors

In the last decade, the role of epigenetics in RA development has started to be unraveled. Epigenetic mechanisms induce heritable variations in gene expression without actual changes in the deoxyribonucleic acid (DNA) sequence (58, 59). In this way, they may help to explain the low concordance rate observed between monozygotic twins (9–15%) (8–10) and the incomplete contribution of genetic factors to the disease (15). Indeed, a recent large epigenome-wide association study found differentially variable methylation signatures in monozygotic twin pairs discordant for RA (60). Additionally, because epigenetic modifications can be induced by external stimuli (e.g., drugs, smoke, diet), they might provide the link between genome and environment interactions (61). The major epigenetic changes include DNA methylation, post-translational histone modifications and non-coding RNAs, all of which have been shown to contribute to RA susceptibility (58, 59).

Differential **DNA methylation** signatures have been described in peripheral blood mononuclear cells (PBMCs) and fibroblast-like synoviocytes (FLSs) of RA patients (62–65). These include global hypomethylation of these cell populations (62, 64), as well as hypo- or hypermethylation of specific promoter regions, which lead to an increase or decrease, respectively, in the transcription of pro- (e.g., *IL-6, IL6-R, CXCL12, CD40L*) or anti-inflammatory [e.g., *CTLA4* in T regulatory (Treg) cells] genes (58, 59, 63, 66, 67). Interestingly, treatment with methotrexate (MTX) has been shown to reverse the global hypomethylation of B cells, T cells and monocytes (64), as well as to restore Treg cell function through demethylation of the *FOXP3* locus (68), highlighting the reversible nature of epigenetic changes and its potential as a therapeutic target. Furthermore, recent epigenome-wide studies have reported several differentially methylated genes in blood samples of RA patients (69). A large study identified two clusters located within (nine genes) or in close proximity (one gene) to the *HLA* locus, suggesting that the genetic risk for RA conferred by this region is, at least partially, mediated by DNA methylation (65). Likewise, other studies applied the same approach in RA and control [osteoarthritis (OA)] FLSs and were able to identify different methylation patterns in key genes involved in RA pathogenesis (70–73). Surprisingly, within RA patients, two separate groups recently identified joint-specific methylome and transcriptome signatures that varied across several joint locations (e.g., hip, knee, MCP), providing a possible explanation for the distinctive articular pattern of the disease (74, 75).

Substantially less is known about the role of **histone modification** in RA. Histones can be modified by processes such as acetylation, methylation, phosphorylation, citrullination, and others, resulting in alterations of chromatin structure and, consequently, gene transcription (58, 59). Most studies have focused on acetylation status in blood and synovial tissue of RA patients, by measuring the expression and balance of the histone-modifying enzymes histone deacetylases (HDACs) and histone acetyl transferases. Both decreases (76) and increases (77–79) in synovial tissue activity and expression of HDACs have been reported, although the latter are likely to be more important and have also been reported in blood (80). Different, sometimes antagonistic, effects of specific HDACs or HDAC classes may contribute to complexity (81, 82). Nonetheless, various studies have demonstrated the impact of HDAC inhibitors in suppressing inflammation [notably, IL-6 and type-I interferon (IFN) responses], angiogenesis, and function and survival of FLSs and macrophages (79–84). Importantly, it was recently demonstrated that smoking, a major environmental risk factor for RA, increases the levels of two key HDACs [sirtuin (SIRT) 1 and SIRT6], again reinforcing the importance of epigenetics for gene-environment interface (85, 86).

**Non-coding RNAs** are yet another mode of epigenetic regulation and include microRNAs (miRNAs, around 22 nucleotides) and long non-coding RNAs (lncRNAs, over 200 nucleotides), both of which have been extensively studied in RA susceptibility, severity and treatment (58, 87, 88). miRNAs are non-coding RNAs that bind messenger RNA (mRNA), leading to its destruction or blocking its translation. Due to this regulation effect on gene expression, they have been the object of significant attention in areas like oncology, metabolic diseases and inflammatory arthritides (87). A wealth of miRNAs have been studied in RA, of which the most established in terms of relevance for RA pathogenesis include miRNA-155, miRNA-146a, miRNA-223 and miRNA124a (58). The earliest and best-studied are miRNA-155 (89) and miRNA-146a (90) which were shown to be increased in many cells (B, T and NK cells, macrophages, FLSs) and tissues (blood, synovial tissue/fluid) of RA patients and to have pleiotropic, but opposite, roles in promoting or suppressing, respectively, several inflammatory, proliferating and bone erosion pathways (58, 87). Specific gene targets of these miRNAs have been validated, confirming their role in RA pathogenesis (58). miRNA-223 and miRNA-124a have also been shown to be increased and decreased, respectively, in RA synovial tissue/fluid, FLSs and blood cells, directly contributing for regulation of osteoclastogenesis, FLS proliferation, and T cell and macrophage-mediated inflammation (58, 91, 92). In recent years, lncRNAs have started to be investigated in RA, due to their function as nuclear and cytoplasmic regulators of gene transcription and mRNA translation (58, 88). Several dozen lncRNAs have been reported to be differentially expressed in RA, and in just over ten the functional role was shown to involve regulation of inflammation and matrix degradation pathways in FLSs, T cells and monocytes (58, 88). The most characterized lncRNA is HOTAIR, which represses the expression of matrix metalloproteinase (MMP)-2 and MMP-13 (93). It was found to be increased in PBMCs of RA patients (94), as well as in FLSs from lower vs. upper extremity joints (74), implicating it as another mechanism involved in RA joint patterning (93).

### Hormonal, Reproductive and Neuroendocrine Factors

Considering the female preponderance in the distribution of RA**, hormonal** and sex-related factors have long been investigated as predisposing to the disease (95). The sex imbalance is commonly attributed to estrogens, generally described as being pro-inflammatory, in opposition to the anti-inflammatory effects of progesterone and androgens, which are decreased in male and female RA patients (96). However, their actions are far more complex and, in fact, estrogens also possess anti-inflammatory properties in a number of cells and tissues (96, 97). The global net effect is likely dependent on other factors such as serum and tissue concentration, predominant cell types and estrogen receptors involved, as well as the reproductive stage (95, 97). These mechanistic aspects are important to understand the conflicting findings reported for a number of hormonal and reproductive factors in the risk of RA. Parity, breastfeeding, pregnancy loss, early menarche, age at first pregnancy, OCs and hormone replacement therapy have all been associated with increased, unchanged or decreased likelihood of development of RA (95). Although in some instances the evidence points slightly more strongly in one direction [e.g., breastfeeding (98) as protective factor in recent meta-analyses], in other cases [e.g., OCs (99) and parity (100, 101)] there is controversy even between separate meta-analyses. Thus, the true effect of such aspects is currently not fully understood, as additional factors such as varying estrogen dosage, differential impact in seropositive/seronegative disease, or interactions with other reproductive or environmental factors may contribute to heterogeneity. On the other hand, situations associated with an abrupt decline in global estrogen load, such as early menopause (102), post-menopausal stage (103), puerperium (104) and anti-estrogen agents [selective estrogen receptor modulators (105) and aromatase inhibitors (105, 106)], have been more consistently identified as risk factors for RA (95). However, a recent robust study failed to confirm an association with tamoxifen or aromatase inhibitors with the risk of incident RA in women with breast cancer (107). Finally, pregnancy itself has been historically associated with both reduced incidence and major clinical improvement of RA during the gestational period, an observation that still stands today and is attributed to the profound hormonal (sharp rise in estrogen and progesterone) and immunological [T helper (Th) 1-to-Th2 shift] maternal changes (104, 108).

Although with more limited evidence, other related risk factors for RA include pre-eclampsia (109) and both low and high birth weight (110, 111). The former was hypothesized to be explained by fetal microchimerism (i.e., the exchange and persistence of fetal cells in maternal circulation), which is increased both in women with pre-eclampsia (112) and with RA (113), where it is thought to mediate maternal acquisition of the SE. On the other hand, birth weight has been shown to have a U-shaped association with decreased adult hypothalamic-pituitary-adrenal (HPA) function (114). As RA patients have decreased cortisol levels and responsiveness (115), birth weight extremes were proposed to contribute to RA through downregulation of the HPA axis, setting it to function at a reduced level (110).

Indeed, ever since the first anecdotal reports of major clinical improvement induced by incident Cushing's disease (116) or focal neurological lesions (117), disturbances in the **neuroendocrine system** and its relation with the immune system have been implicated in RA (118, 119). During systemic inflammation, both the HPA axis and the autonomic nervous system are physiologically activated centrally, in an attempt to suppress peripheral inflammation. This is achieved, respectively, through anti-inflammatory hormones (cortisol, adrenal androgens) and neurotransmitters [norepinephrine (ß receptors), adenosine (A2 receptors) and endogenous opioids (μ receptors)] (118). Several studies have shown that these pathways are dysregulated in RA, leading to a proinflammatory ambient at the joint level and, consequently, to synovitis (120).

Hypothalamic hyporesponsiveness to stress and inflammation was first suggested (115), but a state of relative adrenal insufficiency with inappropriately low levels of cortisol and adrenal androgens upon chronic inflammation has since been established as the key pathogenic mechanism (118, 121, 122). Changes in the circadian rhythm of secretion of cortisol and of proinflammatory hormones melatonin and prolactin, also likely play a role (115, 123, 124). At the synovial tissue level, there is impaired ability to reactivate inactive cortisone (125); increased levels, local synthesis and action of melatonin (126); upregulated aromatase activity with enhanced androgen-to-estrogen conversion and high estrogen-to-androgen ratio (126, 127); and a preponderance of estrogen receptor-ß over estrogen receptor-α (120, 128).

These endocrine imbalances are paralleled by changes in the **sympathetic and sensory nervous systems** that also contribute to RA (118, 119). Loss of synovial sympathetic nerve fibers, probably due to the production of nerve repellent factors by macrophages and FLSs, and preservation of sensory nerve fibers (at a 1: 10 ratio) have been described in the rheumatoid synovium (120, 129). This leads to a proinflammatory environment, due to the predominant effects of the sensory neuropeptide substance P and a shift in sympathetic signaling from anti-inflammatory ß and A2 receptors to inflammatory α and A1 receptors (118). Moreover, there is an increased systemic sympathetic tone, which is thought to be a physiological response to the decreased HPA axis function and that results in the uncoupling of these two systems (119, 130). Overall, defects in physiological neuroendocrine mechanisms contribute to immune system dysregulation and lead to the establishment and perpetuation of RA. This is best exemplified by the effect of psychological stress (131), including childhood traumatic stress (132) and post-traumatic stress disorder (133–135), in predisposing for or aggravating RA (136).

Another important endocrine mediator with immunomodulatory properties is **vitamin D**. The close relationship with the immune system has been revealed in the last 20 years and, currently, vitamin D is known to exert pleiotropic anti-inflammatory effects through direct action in several immune cells (macrophages, dendritic cells, lymphocytes, FLSs) that express the vitamin D receptor (137). This fact, together with the replicated observation that reduced vitamin D levels and vitamin D deficiency were common in RA patients (138), led to the investigation of vitamin D as a potentially protective factor for RA (139). The present situation is still equivocal as large prospective cohort studies have either found (140) or failed to find (141) an inverse association of RA incidence and vitamin D intake. These studies were later meta-analyzed and a 24% risk reduction was observed in high vs. low vitamin D intake (142), although the main issue is that dietary questionnaires are likely not the best method to assess vitamin D status, which can be affected by time fluctuations, sun light exposure and other confounding factors (137). Interestingly, a subsequent study of the same cohort that had negative results for vitamin D intake, reported that higher cumulative ultraviolet-B light exposure, which is the primary source of vitamin D, significantly decreased RA risk (143). Further suggestions of a link between vitamin D and RA come from studies reporting associations with SNPs from the vitamin D pathway genes *VDR* (144) (encoding vitamin D receptor), *GC* (145) (encoding vitamin D-binding protein) and *CYP27B1* (146) (encoding 25-hydroxy-vitamin D-activating enzyme 1α-hydroxylase) (137, 139). Nevertheless, the true role of vitamin D as a protective factor for RA remains unclear and a large randomized controlled trial (RCT) failed to demonstrate a preventive effect of daily calcium and vitamin D supplementation on RA risk (147). Instead, more robust evidence supports the association of vitamin D deficiency and a poor prognosis in RA patients, determined by increased disease activity, functional impairment and poor HRQoL (137–139).

Finally, evidence supporting **obesity** as a risk factor for RA is mounting (6, 148–151). Two recent meta-analyses including up to 13 studies have confirmed a positive association of obesity with RA (pooled RR 1.21–1.31) and a dose-response effect of body mass index (BMI) on RA risk (149, 150). There are also some indications that this association is stronger in women and, potentially, seronegative disease, although in the latter case the two meta-analyses have conflicting results (149, 150). These findings are in accordance with the secular rise in BMI and obesity prevalence at RA presentation observed in the last two decades (148, 152). While obesity may be regarded as a surrogate for other environmental and lifestyle risk factors (see below), its predisposing effect for RA may be explained by metabolic and endocrine mechanisms. Plausible hypotheses include increased adipocyte secretion of proinflammatory cytokines and adipokines, as well as perturbation of sex hormone metabolism, with increase in estrogen levels due to enhanced aromatase-mediated conversion in the adipose tissue (153, 154). Moreover, hyperlipidemia, which is linked to obesity, has been reported to be increased in individuals who develop RA, particularly in women (155–157). In line with this, higher statin use has been shown to be protective of incident RA in large cohort (158) and nested case-control studies (157, 159) (more than 500,000 participants in one study that found a 23% decrease in risk) (159). This effect has been hypothesized to be linked both to lower lipid blood levels and to the anti-inflammatory actions of statins. However, the role of statins as risk factors is still not entirely understood. A case-control study found a contrasting increased risk of RA (though with no cumulative dose trend) (160) and other two very large [*n* > 1,000,000 (161) and *n* > 2,000,000] (162) prospective cohort studies failed to detect an association with incident RA. In one of these studies there was even a potential increase of RA risk in the first year after statin commencement, that progressively returned to normal (161). Discrepancies are likely due to methodological and definition issues, but clearly a definite conclusion cannot yet be drawn.

### Comorbid Host Factors

There have been epidemiological associations of other, apparently unrelated, concurrent diseases with increased future risk of developing RA. This is different from comorbidities affecting established RA patients (e.g., cardiovascular disease, infection, lymphoma, osteoporosis), which occur at higher rates than the general population and have a significant impact in the prognosis of the disease (see below) (2, 163).

**Psychiatric conditions** appear to have a particularly interesting link with RA. As mentioned above, an association between post-traumatic stress disorder and an increased risk of RA has been described in both men (133) and women (134). This has been hypothesized to be related to the previously described dysregulated neuroendocrine-immune mechanisms induced by chronic stress (136). Most recently, depression, a well-known common RA comorbidity found in 15–17% of patients, was also proposed as a risk factor for RA, suggesting a bidirectional relationship (164–166). This observation follows the publication of three large longitudinal cohort studies that identified depression to confer a 28–65% increase in the risk of developing RA (167–169). Interestingly, one of the studies showed that the use of antidepressants among depressed patients was protective of RA development [hazard ratio (HR) 0.74, 95% CI 0.71–0.76] (168), whereas another study found it to be associated with subsequent seronegative RA (HR 1.75, 95% CI 1.32–2.32) (169). Novel insights into the pathogenesis of depression, suggesting prominent systemic inflammatory mechanisms, were proposed as a possible explanation for the association with RA (165). Similar, recently identified relations with other rheumatic (psoriatic arthritis), gastroenterological (inflammatory bowel disease) or dermatological (alopecia areata, vitiligo) immune-mediated diseases, further support this hypothesis (165).

In contrast, a puzzling consistent negative association has been recognized with schizophrenia for over eight decades now (170, 171). These epidemiological data were revisited in two updated meta-analyses also including the latest studies, which confirmed the significant protective effect of schizophrenia for the development of RA (OR 0.48–0.65) (172, 173). A possible infectious cause was first proposed as an explanation for this intriguing observation, but recent data have strengthened the genetic-immunologic theory (170, 174, 175). Studies have demonstrated a negative SNP-genetic correlation between the two conditions (174) and identified pleiotropic SNPs in established HLA risk genes that differentially contributed for RA and schizophrenia (i.e., based on the specific allelic variant within the same gene) (175). Although still a matter of debate, these interesting complex genetic mechanisms help to explain how a given disease can reduce the chance of developing another seemingly unconnected condition.

**Atopy and allergic diseases** (e.g., asthma, allergic rhinitis, atopic dermatitis) were initially suggested to be negatively associated with the risk of RA, an observation that was hypothesized to be related to the predominance of Th2-dependent pathways, as opposed to a Th1 phenotype in RA (176). Since then, several epidemiological studies have rebutted this view and reported an increase in RA incidence in allergic populations (177–180). Although the literature is controversial, most high-quality population-based cohort studies point toward a positive, rather than negative, association between atopy and RA (177). This has been reinforced by large recent studies (181–183), and linked to possible shared genetic (e.g., *HLA-DRB1, IL-6R, CD40L*), immunological [e.g., natural killer (NK), Th1 and Th17 cells, TNF] and environmental (e.g., smoking) mechanisms (16, 177). In addition, **respiratory diseases**, both acute and chronic, and of the upper or lower airway tract, have recently been associated with an increased risk of seropositive and seronegative RA (184). The association, though, was limited to non-smokers, suggesting possible separate, or complementary, pathogenic pathways in smoking and respiratory disease. Other studies have also confirmed the association of chronic obstructive pulmonary disease with subsequent RA (179, 183, 185, 186).

Several other diseases have been identified as risk factors for incident RA, most notably other non-rheumatological **immune-mediated diseases**, such as autoimmune thyroid disease (i.e., Hashimoto thyroiditis and Graves' disease) (187, 188), type 1 diabetes mellitus (187, 189), alopecia areata (190), vitiligo (191), inflammatory bowel disease (192) and, possibly, multiple sclerosis (193) (less robust evidence) (187). Various common genetic risk determinants have been identified and other host and external factors are also likely to be important (16, 17, 38, 45, 50, 61, 188, 189). Interestingly, despite being an important RA comorbidity, one population-based case-control study has associated type 2 diabetes mellitus with increased risk of incident RA, (194) although no effect had been reported in an earlier similar study (189).

Finally, large longitudinal cohort studies have recently suggested that **sleep disorders** (195), including both obstructive sleep apnea (195, 196) and non-apnea sleep disorders (195, 197), could be additional risk factors for RA. The influence of sleep disturbances on immune dysregulation has also been proposed as a possible explanation for the 91% increased risk of RA found in patients with migraine (198), highlighting the complex multifactorial nature of the disease.

## Environmental Factors

Although the data above support a large impact of host on the development of RA, the environment also plays a fundamental role in determining the ultimate risk of disease. In fact, extrinsic factors have been identified to interact with at-risk subjects and confer a multiplicative increase in the likelihood of developing RA (Figure 1). Environmental factors can be roughly grouped into four categories: airborne exposures, notably including smoking; microbiota and infectious agents; diet; and socioeconomic factors, including occupational and recreational exposures. Extensive data are available directly implicating these numerous aspects in the etiology of RA.

### Smoking and Other Airborne Exposures

The recognition of the lung as a major site of early pathogenic events has been one of the great breakthroughs in the understanding of the disease and is well-exemplified by the strong association of several airborne noxious agents with RA (199, 200).

**Smoking** is the most important of such exposures and has been established as one of the main risk factors for the development of RA (199). Its association with RA has been extensively replicated since the first description more than three decades ago (201), and smoking is currently thought to explain 20–25% of overall RA risk and up to 35% of ACPA-positive RA (202, 203). A meta-analysis of 16 studies estimated an overall OR for ever smoking of 1.40 (95% CI 1.25–1.58) (204). The effect was stronger for RF-positive RA (OR 1.66, 95% CI 1.42–1.95) and in men (OR 1.89, 95% CI 1.56–2.28), with a multiplicative interaction (RF-positive men: OR 3.02, 95% CI 2.35–3.88) (204). Moreover, there is a clear dose-response relationship, with significantly higher risks for current or heavy vs. past or light smokers, respectively, and a linear increase in risk with smoking pack-years (203, 204). Accordingly, smoking cessation was shown to progressively decrease the risk of RA development, returning to that of never smokers after a period of 20–30 years (202, 203, 205). This is, therefore, a practical advice that should be routinely given to patients, especially to those at higher risk of developing the disease. Passive smoking should also be avoided, as studies have additionally suggested a possible link with prenatal (111, 206, 207), childhood (207, 208) and adult (203) passive exposure to cigarette smoke.

A key discovery was the gene-environment interaction of smoking with SE alleles and seropositive RA, with a multiplicative dose effect of both smoking load (e.g., OR 6.3, 12.0, 24.6, and 37.6 in homozygotic SE carriers with 0, <10, 10–19 or ≥20 pack-years, respectively) and SE risk alleles (e.g., RR 21.0, 6.5, and 1.5 in ever smokers carrying two, one or no copies of SE genes, respectively) (199, 202, 209). All of these epidemiological observations have provided the basis for a novel model of RA pathogenesis, in which smoking is responsible for *in situ* protein citrullination (i.e., the conversion of arginine to citrulline) in the lungs of SE-positive individuals, with the subsequent generation of ACPAs and, eventually, RA (199, 209). Subsequent demonstration that both activity and expression of the citrullination enzyme PAD2 are increased in the bronchioalveolar compartment of smokers (210) and that, after citrullination, peptides such as vimentin or fibrinogen bind specifically to SE-containing HLA molecules and induce the emergence of autoreactive T cells (211), further supported this hypothesis (209). In line with it, cigarette smoke, rather than tobacco or nicotine *per se*, seems to be crucial in the process, as it leads to chronic airway inflammation (200), whereas non-inhaled moist snuff tobacco does not increase the risk of RA (212). Moreover, the RA risk associated with smoking has been shown to be influenced by SNPs and deletions in genes encoding enzymes involved in the detoxification of smoke carcinogens [e.g., glutathione S-transferases (213, 214), N-acetyltransferases (215) or heme-oxygenase (214)], again demonstrating the importance of smoke-induced changes in the respiratory airway. Finally, carbamylation (i.e., the conversion of lysine to homocitrulline) has also been reported to be associated with smoking, generating novel autoantigens that are targeted by the RA immune-specific response (216). Indeed, anti-carbamylated protein antibodies have been described in the serum of pre-RA subjects, and predict the development of RA (217).

Several other inhaled agents are thought to exert similar harmful effects and increase the risk of RA (200). The first to be recognized and best studied is **silica**, a common occupational exposure (e.g., mining, construction or ceramic industries) that is independently associated with RA (OR around 2–3) (218, 219). Similarly to smoking, it is specifically associated with ACPA-positive RA and both exposures have an additive effect (OR 7.36 in silica-exposed smokers), which increases with pack-years of smoking (220, 221). Additionally, **textile dust** (OR 2.8, 95% CI 1.6–5.2, similar for ACPA-positive and ACPA-negative RA, but with an interaction with HLA-SE in the former: OR 39.1, 95% CI 5.1–297.5) (222) and **inorganic dust** (e.g., asbestos, cement) (223) were also linked to RA (224). In contrast, despite a few contradicting reports (225), ambient **air pollution** does not seem to be a consistent risk factor for RA (226).

### Microbiota and Infectious Agents

The “infectious hypothesis” has long been proposed as a likely explanation for the development or triggering of RA (227). The decline in RA incidence observed in various populations following the improvement in health and sanitary conditions was one of the main facts indirectly supporting this possibility (228–230). It was reinforced by epidemiological and translational studies directly involving specific viruses, bacteria and other microbial agents, that could contribute to RA through non-specific immune activation, molecular mimicry or other mechanisms (227, 231). However, after decades of research, no single infectious agent has been consistently identified to be the cause or to increase the risk of RA (231). Notably, in the last years, the aforementioned mucosal immunity, together with oral/intestinal dysbiosis and chronic infections have been closely implicated in the etiology and pathogenesis of RA (199, 232).

**Periodontitis**, the main of such factors, results from dysbiosis of the oral microbiota and has been associated with increased risk of RA (233). The relationship between both diseases is bidirectional (i.e., RA patients also have higher likelihood to develop periodontitis) and profound, as they share similar genetic (e.g., HLA-SE alleles) and environmental (e.g., smoking, nutrition) risk factors and both lead to chronic inflammation, bone erosion and tissue destruction (233, 234). *Porphyromonas gingivalis* (*P. gingivalis*) is a major cause of periodontitis and the most important agent that has been specifically associated with RA (233). Its involvement is not just circumstantial but an etiologic role has been proposed through mechanisms similar to those previously described for smoke (235). In fact, *P. gingivalis* is unique in that it has its own PAD, that can cause chronic citrullination of bacterial and host proteins (236), leading to breach of immune tolerance, ACPA production and, eventually, through molecular mimicry and/or epitope spreading, culminating in RA (233, 235). This theory is supported by several studies demonstrating PAD activity, protein citrullination and ACPA generation in the inflamed periodontium (237–239); gingival citrullination patterns in periodontitis that mirror those seen in the rheumatoid joint (239); increased ACPA titers in periodontitis patients (240); cross-reactivity between RA-specific anti-human α-enolase ACPAs and *P. gingivalis* (241); and increased antibodies against *P. gingivalis* in at-risk subjects and in patients with periodontitis, pre-RA, RA and ACPA-positive RA, that additively interacted with smoking and *HLA-DRB1* (233, 242–244). Of note, a recent study further demonstrated that oral dysbiosis, enriched in *P. gingivalis*, was present even in periodontally healthy sites of ACPA-positive at-risk individuals (245). In addition, another pathogenic periodontitis agent, *Aggregatibacter actinomycetemcomitans* (*A. actinomycetemcomitans*), has been directly implicated in RA through different mechanisms involving neutrophil-mediated citrullination (239). Nonetheless, despite all of the rationale and evidence supporting the periodontitis-RA link, it should be noted that the more robust, largest, population-based prospective studies have either failed to demonstrate an association of periodontitis with incident RA (246–248) or did so with a small effect size (OR 1.16–1.17) and without adjustment for major confounding factors such as smoking (249, 250). Whether this is the result of methodological and disease-definition issues or actually reflects a smaller role of periodontitis in RA risk is unclear.

Growing interest has been devoted in the last decade to **gut microbiota** and its role in immune homeostasis and the development of disease (251). Intestinal dysbiosis has been linked to a number of inflammatory rheumatic diseases, including RA (251, 252). Evidence supporting this association firstly stems from animal studies demonstrating that microbial flora is indispensable for the development and aggravation of experimental arthritis (232, 251, 253). In addition, human studies reported changes in the composition of gut microbiota of RA patients, with decreased microbial diversity, enrichment of *Prevotella copri, Lactobacillus* spp. and *Clostridium* spp. and decrease in *Bacteroides* spp. and *Haemophilus* spp (232, 251, 253–257). Interestingly, a comprehensive metagenome-wide association study demonstrated remarkable concordance between fecal and oral dysbiosis patterns, which could differentiate RA patients from controls with remarkable accuracy [area under the curve (AUC) 0.94 and 0.87, respectively] (232). It is important to mention that all clinical association studies have a cross-sectional design and, thus, do not clearly imply an etiologic role. Nevertheless, a direct causal effect has been suggested by recent studies showing that RA gut dysbiosis induces activation of autoreactive T cells (253) and that HLA-DR-presented *Prevotella copri* peptides can generate RA-specific Th1 and Th17 responses (258). In line with this, *Prevotella* spp has been shown to be enriched in subjects at risk for RA, further implicating intestinal dysbiosis in the pathophysiology of RA (257, 259). Moreover, the fact that oral and gut microbiome dysbiosis patterns of new-onset untreated RA patients were correlated with disease activity and improved with conventional synthetic DMARD (csDMARD) treatment, also provides indirect evidence of pathogenicity (232).

As mentioned, external **infectious agents** have been implicated as risk factors for RA for decades (227). Besides the discussed relation with the decrease in RA incidence over time, putative mechanisms linking infection and RA include molecular mimicry, epitope spreading, B cell amplification/proliferation, non-specific inflammatory activation and superantigens (260). Several studies supported this concept by demonstrating increased prevalence of microbial-specific antibodies in the serum of RA patients and identifying bacterial/viral proteins or genetic material in rheumatoid synovial fluid and/or tissue (231). Commonly reported agents include Epstein-Barr virus (EBV), cytomegalovirus (CMV), parvovirus B19, rubella virus, mycoplasma, *Proteus mirabilis, Escherichia coli*, hepatitis B/C virus, *Borrelia burgdorferi* (Lyme's disease), Chikungunya virus, and others (5, 227, 231, 260). However, the epidemiological associations are inconsistent and the overall study quality is poor (260). This was revealed in a recent meta-analysis of 48 studies that concluded that only parvovirus B19 (OR 1.77, 95% CI 1.11–2.80), hepatitis C virus (OR 2.82, 95% CI 1.35–5.90) and, possibly, EBV [anti-VCA (OR 1.5, 95% CI 1.07–2.10) and anti-EA (OR 2.74, 95% CI 1.27–5.94) but not anti-EBNA antibodies], but not hepatitis B, CMV or other viruses were associated with RA (260). A previous meta-analysis of 23 studies of EBV seroprevalence had failed to demonstrate an increased risk of RA, especially when only higher quality studies were considered (261). These findings are in disagreement with extensive experimental research implicating EBV in RA through potent B cell stimulation, molecular mimicry (including cross-reactivity with citrullinated host antigens and recognition of anti-EBV antibodies by HLA-DR), increased blood and synovial tissue viral load and impaired viral-specific T cell response (231, 262). The association therefore remains equivocal for most, if not all, infectious agents. An important confounding issue is the fact that these infections *per se* can cause RA-like polyarthritis in the acute phase (e.g., parvovirus B19, rubella), chronic polyarthralgia/polyarthritis following the disease (e.g., Chikungunya, Lyme's disease) and even generate ACPAs or RF (e.g., hepatitis C virus), thus making it harder to ascertain case definition (231, 260). Moreover, limited available evidence failed to demonstrate temporal or spatial clustering of incident RA that could be related to a common infectious exposure (263). Surprisingly, recent self-reported bacterial urogenital and gastrointestinal, but not respiratory, infections were found to be associated with a decreased risk of incident RA in a large population-based case-control study (264). This effect was hypothesized to be due to antibiotic- and/or infection-induced changes in the microbiota that could, in this case, be protective.

Nonetheless, taking all the data into account, it is likely that, as with other environmental factors, infectious agents have some kind of role in priming, triggering or potentiating disturbed immune mechanisms in a genetically predisposed host. Novel evidence supporting this concept comes from an important study that elegantly demonstrated a mechanism mediated by protective/predisposing HLA alleles, through which infection (and microbiota) can influence the risk of ACPA-positive RA (265). Cross-recognition by CD4^+^ T cells of an epitope (DERAA) that is found both in synovial citrullinated vinculin and in several microbes (including gut bacteria such as *Lactobacillus*, enriched in RA) (232, 254, 255) and which is presented by predisposing HLA-DQ molecules (HLA-DQ5, DQ-7.3 and DQ8, all in tight linkage disequilibrium with HLA-DR SE alleles), leads to increased ACPA production and, eventually, RA (265).

### Diet

The epidemiological association of dietary factors with RA has been extensively studied. In spite of the difficulties in accurately assessing patient nutritional behavior before RA onset and isolating the effect of a given food, drink or nutrient, some findings are consistent. Importantly, they also provide clues on RA etiology and pathogenesis, as shown by the influence of the modulation of the intestinal microbiome by diet on the risk of developing RA (251, 252).

Low-to-moderate **alcohol** consumption is protective of RA development. A meta-analysis that included only cohort or nested case-control studies (*n* = 8; 195, 029 participants), reported a 14% decrease in the risk of RA (RR 0.86, 95% CI 0.78–0.94) (266). The effect was dependent on dose (J-shaped non-linear trend, with greater benefit for 9g/day vs. 3 or 12g/day), time (17% reduction if consistent intake for ≥10 years) and sex (19% reduction in women) and unrelated to beverage type (266). Alcohol-induced downregulation of the immune response and proinflammatory cytokine production has been proposed as an explanation for this observation (267). Most recently, a population-based study confirmed the protective nature of alcohol in both ACPA-positive and ACPA-negative RA and reported an additive interaction with HLA-SE and smoking for development of the former (OR 25.3, 95% CI 17.7–36.2 for never-drinkers, ever smokers, HLA-SE-positive patients) (268). The mechanism justifying this interaction is currently unclear.

Similarly, general **healthy eating behaviors** have also been associated with decreased risk of RA (251, 252). Long-term adherence to a healthier diet (assessed through a standard dietary quality score) in women from the Nurses' Health Study (*n* = 169, 989) was protective of younger-onset (≤ 55 years-old) seropositive RA development (HR 0.60, 95% CI 0.51–0.88) (269). Subgroup analysis revealed that lower red/processed meat (OR 0.58, 95% CI 0.43–0.79) and sodium (OR 0.65, 95% CI 0.44–0.98) consumption were associated with a significant decrease in RA risk (269). A previous analysis of the same study had also associated daily consumption of sugar-sweetened soft drinks with a 63% increased risk of seropositive RA, which was higher in RA starting after the age of 55 (HR 2.64, 95% CI 1.56–4.46) (270). Similar findings implicating high red meat intake as a risk factor for RA had been reported in another prospective cohort (271). Interestingly, sodium has been shown to interact with smoking to increase RA incidence only in smokers (OR 2.26, 95% CI 1.06–4.81), particularly that of ACPA and/or HLA-SE-positive disease (272).

In contrast, an important component of a healthy diet is the consumption of food rich in **polyunsaturated oils** (e.g., omega-3 fatty acids), such as fish and olive oil. Most evidence supports the protective role of fish (273, 274), omega-3 and omega-6 fatty acids (274, 275), and olive oil (251, 276). Although a few studies failed to find a beneficial association with these foods, compelling mechanistic evidence has been recently provided by a nested-case control study that demonstrated an inverse relation between RF- (OR 0.27, 95% CI 0.10–0.79) and ACPA-positivity (OR 0.42, 95% CI 0.20–0.89) in SE-positive individuals at risk for RA (251, 277). Moreover, RCTs have demonstrated that fish oil improves pain outcomes and clinical response to csDMARDs, corroborating a possible role in RA pathogenesis (251, 278).

**Fruits and vegetables** are a hallmark of a balanced diet and a major source of fiber and antioxidant elements such as vitamin C. Both have been associated with a decreased risk of RA in robust prospective studies, (276, 279, 280) although data from another large cohort could not confirm these findings (269). Nonetheless, the overall picture is clear in pointing toward a trend for a protective role of healthier nutritional behaviors in RA development. A Mediterranean diet, usually richer in fruits, vegetables, olive oil and fish, has been proposed as a possible explanation for the North-to-South gradient of RA seen in Europe, complementary to other genetic and environmental (e.g., sun exposure) factors (6, 251, 252). As an intervention, it has been shown to improve RA inflammation, pain and function (251). However, a positive effect of adherence to a Mediterranean diet could not, thus far, be demonstrated (281). This could be due to statistical and epidemiological issues, as, for example, a follow-up analysis of one of such negative studies, with a larger sample size and longer follow-up did demonstrate a benefit of healthier diet, close to the Mediterranean diet definition, in RA risk (269).

Finally, **coffee, tea and caffeine** have been inconsistently associated with RA. A meta-analysis including five studies (2 cohort and 3 case-control) and 134,901 participants found an increased risk with total coffee consumption (RR 2.43, 95 % CI 1.06–5.55) and no association with tea intake (282). Subgroup analyses investigating cohort studies, caffeinated-decaffeinated coffee, caffeine dose or seronegative RA were not significant, but a homogeneous modest association was seen with seropositive RA (RR 1.33, 95% CI 1.16–1.52). More recently, another large prospective cohort study (*n* = 76,853) found no increased RA incidence with coffee (caffeinated or decaffeinated) consumption, whereas caffeinated tea intake conferred a 40% increase in risk (HR 1.40, 95% CI 1.01–1.93) (283). This is in disagreement with the meta-analysis and most previous studies and, as such, the link between coffee, tea and RA remains equivocal.

### Socioeconomic and Other Environmental Factors

A lower **socioeconomic status** seems to increase RA risk, although it probably has a stronger link with poor disease outcome (5, 6). Several studies have demonstrated that a lower level of education is independently associated with RA, particularly with seropositive disease (111, 284–286). Although earlier reports did not find a significant effect of education or other socioeconomic factors on RA risk (287, 288), the associations observed in the positive studies could not be attributed to smoking or other known socioeconomic or lifestyle factors. Moreover, low childhood (i.e., parental) household education and other poor early life socioeconomic status (food insecurity, young maternal age) have also been linked to greater development of adult RA, further supporting the positive epidemiologic observations (111). These studies suggest that socioeconomic deprivation may be an identifiable risk factor for RA, possibly through unmeasured environmental exposures (e.g., infections, low quality diet).

Additionally, a low socioeconomic status may also be more common in individuals with manual, **blue collar jobs**, which, likewise, have been associated with increased RA incidence (185, 224, 285, 289, 290). This association is also seen with paternal occupation (110) and may be related to various factors. First, many blue collar jobs are associated with exposure to silica, inorganic dust, textile dust and other respiratory harmful agents that, as previously discussed, are important RA risk factors (218, 222–224). Second, other blue collar professionals such as auto mechanics and farmers frequently deal with mineral oil (289) and pesticides (291, 292), respectively, both of which have been associated with RA. Interestingly, in two large women cohorts, common direct and indirect (i.e., by others, such as the spouse) pesticide exposure have been reported in adulthood (292), as well as during childhood (293), with a dose-response effect. Third, prolonged repetitive physical workload, typical of blue collar jobs, was recently revealed to be associated with an increased risk of both ACPA-positive and ACPA-negative RA, with an interaction with HLA-SE in the former (290). Fourth, a curious complementary study demonstrated that working in a cold environment increased the odds of developing RA by 50%, both ACPA-positive (60%) and ACPA-negative (40%), also with a dose-response relationship (for indoor work) and an additive interaction with another environmental work-related factor, repetitive hand/finger movements (294). Finally, other **work-related factors** such as work stress, conflict at work and shift work, have also been shown to increase the risk of RA (124, 131).

In contrast to professional activity, two large separate prospective cohort studies [*n* = 30,112 (295) and *n* = 113,366 (296)] have recently shown that higher levels of **recreational physical activity** significantly decrease the risk of incident RA by up to 33–35% (295, 296). This effect was found to be cumulative throughout life and was only partially explained by a decrease in BMI, being possibly linked to the anti-inflammatory properties of exercise (295, 296).

## Discussion

Overall, the body of evidence regarding the etiology of, and risk factors for, incident RA that is available thus far is robust and allows for better understanding of the disease. RA can be regarded as the prototypical multifactorial immune-mediated disease. Multiple susceptibility genes, subjected to expression regulation *via* epigenetic mechanisms, significantly modulate the individual risk of disease. Concomitant hormonal and neuroendocrine determinants, together with comorbid conditions, further determine the likelihood of incident RA. Subsequently, throughout a subject's life, external environmental factors continuously interact with the predisposed host, slowly adding additional breaches to the immune tolerance barrier. Over time, this complex interplay gives rise to multiple cellular and molecular pathophysiological changes that culminate in the crumbling of the entire defense structure against self-aggression. Ultimately, a particular event triggers the final steps that lead to clinically evident overt disease.

A special word is warranted for the impact of cohort studies in the identification of such risk factors. Indeed, robust large-scale longitudinal prospective long-term cohort studies, such as the Nurses' Health Study (297) or the Women's Health Initiative (298), have been instrumental in the unraveling of the relationship between host and environmental factors and incident RA. Although difficult to develop and carry out, requiring extensive investment of time, funds, and effort, these are the most powerful studies to investigate risk factors of a given disease, greatly limiting potential biases of other, more accessible, and feasible study designs, such as cross-sectional, case-control or retrospective cohort studies. As an example, among many other findings, the Nurses' Health Study (NHS) has provided high-quality evidence of the association between diet (299, 300), smoking (205, 207, 299), UV light (143), hormonal/reproductive factors (103, 301), obesity (302), physical activity (296) or depression (169), and RA development. Just as the landmark Framingham Heart Study was pivotal in determining the major risk and protective factors of heart disease (e.g., diet, smoking, exercise, aspirin), saving countless lives at a global level, so too can similar large-scale prospective studies accurately inform on the risk of RA.

Besides providing valuable clues into the etiology of RA, additional insights can be gained from such epidemiological studies. The realization that genetic factors contribute to around one half to one third of RA risk, and that most of the genes implicated are directly linked to the immune system puts immune dysregulation at the core of disease pathogenesis. Moreover, the prominence of the MHC region as the main genetic risk factor, and the importance of HLA-SE molecules as key determinants of disease, confirm that antigen presentation-driven mechanisms are pivotal early pathophysiological steps. The synergic interaction between these genetic variants and environmental factors such as smoking, microbiota and diet bring additional clarity to the events taking place outside the joint. Epigenetic changes, possibly induced by external stimuli, regulate the expression of crucial genes and further determine the final risk of disease. Notably, modulating gene expression through epigenetic regulators is an attractive groundbreaking therapeutic avenue being currently pursued.

On a different level, this knowledge and understanding paves the way for an entirely novel field in RA, which is that of disease prevention (303). In fact, we are currently in the early steps of the path toward what may become, on a short-to-medium term, a new paradigm in medicine in general and rheumatology in particular: being able to correctly identify high-risk individuals at a broad population-level, and institute concrete non-pharmacological and pharmacological interventions aimed at preventing, or delaying, the onset of RA. In this regard, the first step is to stratify subjects according to risk of progression to clinically evident RA (304). Pinpointing those at high risk of developing RA, such as first-degree relatives of patients with RA, ACPA/RF-positive asymptomatic individuals, or patients with clinically suspicious arthralgia, is particularly relevant. Based on the evidence discussed above on the modifiable factors that are strongly associated with RA development, there is rationale to make specific recommendations to these subjects, which may hamper the risk of disease. These high-risk individuals may be advised to stop smoking, maintain a proper oral health, address concomitant conditions such as periodontitis, depression and obesity, and promote a healthy lifestyle, focused on a balanced Mediterranean diet, regular exercise, and stress-limiting activities. This requires the involvement of both rheumatologists and family physicians, who play a central role in health promotion and patient education. Unfortunately, it seems that this is not performed in clinical practice as commonly as desirable (305). A RCT has demonstrated that a personalized education for the risk of RA is more effective in improving healthy behaviors than standard patient education (306). However, it should be noted that these recommendations are mostly supported by epidemiological evidence, which is limited by nature. For instance, an analysis of the NHS has found that smoking cessation was associated with a decreased trend for incident RA (205). Also, while obesity has been associated with RA development, and weight loss is therefore recommended, a prospective study did not show an effect of bariatric surgery in the risk of incident RA. Unfortunately, RCTs of lifestyle interventions, which are needed to firmly establish preventive strategies, are mostly missing. An alternative approach involves pharmacological intervention in at-risk patients. This idea has recently been tested in several clinical trials. The PRAIRI study provided proof-of-concept that such a strategy could be useful, by demonstrating that a single infusion of B cell-depleting rituximab delayed the onset of arthritis in at-risk individuals (307). Other studies are investigating a similar effect with treatments such as abatacept, methotrexate or hydroxychloroquine (303, 308). On the opposite direction, a RCT of ACPA-positive subjects with inflammatory arthralgia did not demonstrate a protective effect of two dexamethasone administrations in progression to RA (309). Similar findings were reported by a systematic literature review and meta-analysis investigating the impact of glucocorticoids, csDMARDs or biologic DMARDs for the prevention of RA in at-risk individuals without arthritis (310). Hopefully, as more data accrues, we will be able to provide additional counseling that can have a potential impact in reducing RA incidence or, at least, delaying its onset.

In summary, over the past decades there has been tremendous progress concerning the etiology and risk factors for the development of RA. While several questions remain unanswered, there is now a clearer notion of the dynamic between host and environmental factors, which sets the key pathogenic events in motion and eventually leads to a step-wise preclinical phase, where mucosal breach of tolerance is followed by systemic autoimmunity and inflammatio, ultimately targeting the articular compartment. This so-called early arthritis stage represents a window of opportunity where it may still be possible to intervene and prevent, or delay, the onset of overt disease. Novel study paths in this field have recently emerged and are likely to bring relevant contributions in the future, improving our understanding of the etiology, risk, and pathogenesis of RA. Ultimately, this will translate into better preventive and therapeutic strategies that will improve the lives and outcomes of patients with RA.

## Author Contributions

Both authors have contributed to study conception and design, collected and analysed the data and drafted the manuscript. Both authors have critically reviewed the manuscript for important intellectual content, and have read and approved its final version.

## Funding

VCR work was funded by Fundação para a Ciência e Tecnologia (Interno Doutorando Bursary reference SFRH/SINTD/95030/2013); European League Against Rheumatism (EULAR Scientific Training Bursary 2014); and Sociedade Portuguesa de Reumatologia (Fundo de Apoio à Investigação da SPR 2015 & 2016, to VCR).

## Conflict of Interest

The authors declare that the research was conducted in the absence of any commercial or financial relationships that could be construed as a potential conflict of interest.

## Publisher's Note

All claims expressed in this article are solely those of the authors and do not necessarily represent those of their affiliated organizations, or those of the publisher, the editors and the reviewers. Any product that may be evaluated in this article, or claim that may be made by its manufacturer, is not guaranteed or endorsed by the publisher.
